# A LabVIEW^®^-based software for the control of the AUTORAD platform: a fully automated multisequential flow injection analysis Lab-on-Valve (MSFIA-LOV) system for radiochemical analysis

**DOI:** 10.1007/s10967-017-5282-2

**Published:** 2017-05-22

**Authors:** Donato Barbesi, Víctor Vicente Vilas, Sylvain Millet, Miguel Sandow, Jean-Yves Colle, Laura Aldave de las Heras

**Affiliations:** Joint Research Centre, Directorate for Nuclear Safety and Security, European Commission, 76125 Karlsruhe, Germany

**Keywords:** Automation, Flow injection analysis, LabVIEW

## Abstract

A LabVIEW^®^-based software for the control of the fully automated multi-sequential flow injection analysis Lab-on-Valve (MSFIA-LOV) platform AutoRAD performing radiochemical analysis is described. The analytical platform interfaces an Arduino^®^-based device triggering multiple detectors providing a flexible and fit for purpose choice of detection systems. The different analytical devices are interfaced to the PC running LabVIEW^®^VI software using USB and RS232 interfaces, both for sending commands and receiving confirmation or error responses. The AUTORAD platform has been successfully applied for the chemical separation and determination of Sr, an important fission product pertinent to nuclear waste.

## Introduction

Trends in analytical chemistry aim at the development of integrated and automated analytical platforms able to perform different steps of the analytical procedure. This results in increased reproducibility and repeatability. Furthermore, the necessity to change the system hardware is common as research and development needs proceed, therefore flexibility is of great importance for the automation of such platforms [1].

In the nuclear field, the development of automated multi-purpose platforms for performing safe and fast determination of radionuclides provides a significant improvement when monitoring operations on-site and/or in situ yielding more effective methods than traditional sampling coupled to manual laboratory analyses. Flow analysis techniques are a major step forward in automating radiochemistry procedures that incorporate extraction and pre-concentration steps, allowing the development of rapid, sensitive and selective methods for the determination of hard to detect radionuclides such as ^90^Sr, ^99^Tc, ^129^I and alpha emitters with high reproducibility. Even though the development and application of automated methods based on flow techniques present a great advance in the nuclear field, most of them are only partially automated and only few are fully automated incorporating on-line detectors.

LabVIEW^®^ is a software tool compiled and commercialized by the National Instruments Corporation. It can be used to develop sophisticated systems both for industry and research [2–6] using its intuitive graphical block diagram, which resembles a wired flow chart [7]. Typically different (sub)VI, each of them performing specific duties, are wired together to perform complex operations. Unlike text based programming languages which need long development and contribution from external experts for maintenance or expansion, LabVIEW^®^ allows preservation of know-how just by transferring software responsibility within the institution.

In recent years, published papers describe such LabVIEW^®^-based software for the automation of specific analytical systems such as flow injection analysis (FIA) [8], sequential injection analysis (SIA) [9] or capillary electrophoresis (CE) [1]. Applications include automatic liquid handling, treatment, separation and, in some cases, detection of the interest analyte. Lab-on-Valve (LOV) devices are programmable flow-based platforms with different coupling modes and high versatility [10]. Such devices allow the automatic separation and preconcentration of the analytes prior to detection thus increasing reproducibility and repeatability and improving sensitivity [11, 12].

The AUTORAD platform is based on a LOV integrated into a multi-syringe flow injection analysis (MSFIA) system enhanced with bed injection (BI), and interfacing an Arduino^®^-based device triggering multiple detectors providing a flexible and fit for purpose choice of detection system [13]. The choice of the detection technique depends on the analyte properties and desired feature of the system. The developed platform and software is able to work with ICP-MS and radio flow detectors, thus improving the flexibility and allowing direct comparison between such detectors. Furthermore, drawbacks of the detection system, such as matrix effects or interferences, can be mitigated using both detectors increasing the versatility of the platform.

Radio flow detection is a versatile and high sensitive technique suitable for many applications [14–17]. The radioactivity detected is directly proportional to the concentration of the analyte according to its speciation. Online β detection allows full automation over a large working range. ICP-MS has shown applicability in quick and accurate determination of long-lived and environmental relevant radionuclides. The working principles are described elsewhere [16, 18, 19].

This paper describes the development and implementation of LabVIEW^®^-Based software for the automatic control of the AUTORAD platform. The possibilities of the resulting control platform are demonstrated by successfully applying for the chemical separation and determination of Sr, one of the most pertinent elements in nuclear waste.

## Experimental

The LOV was fabricated in house from methacrylate and includes eight integrated 16 mm length microchannels: Seven with 1.5 mm i.d. and the column channel with 3.2 mm i.d.. The system is mounted on a Cheminert Selection Valve C25 (Valco Instruments Co. Inc.).

**Fig. 1:**
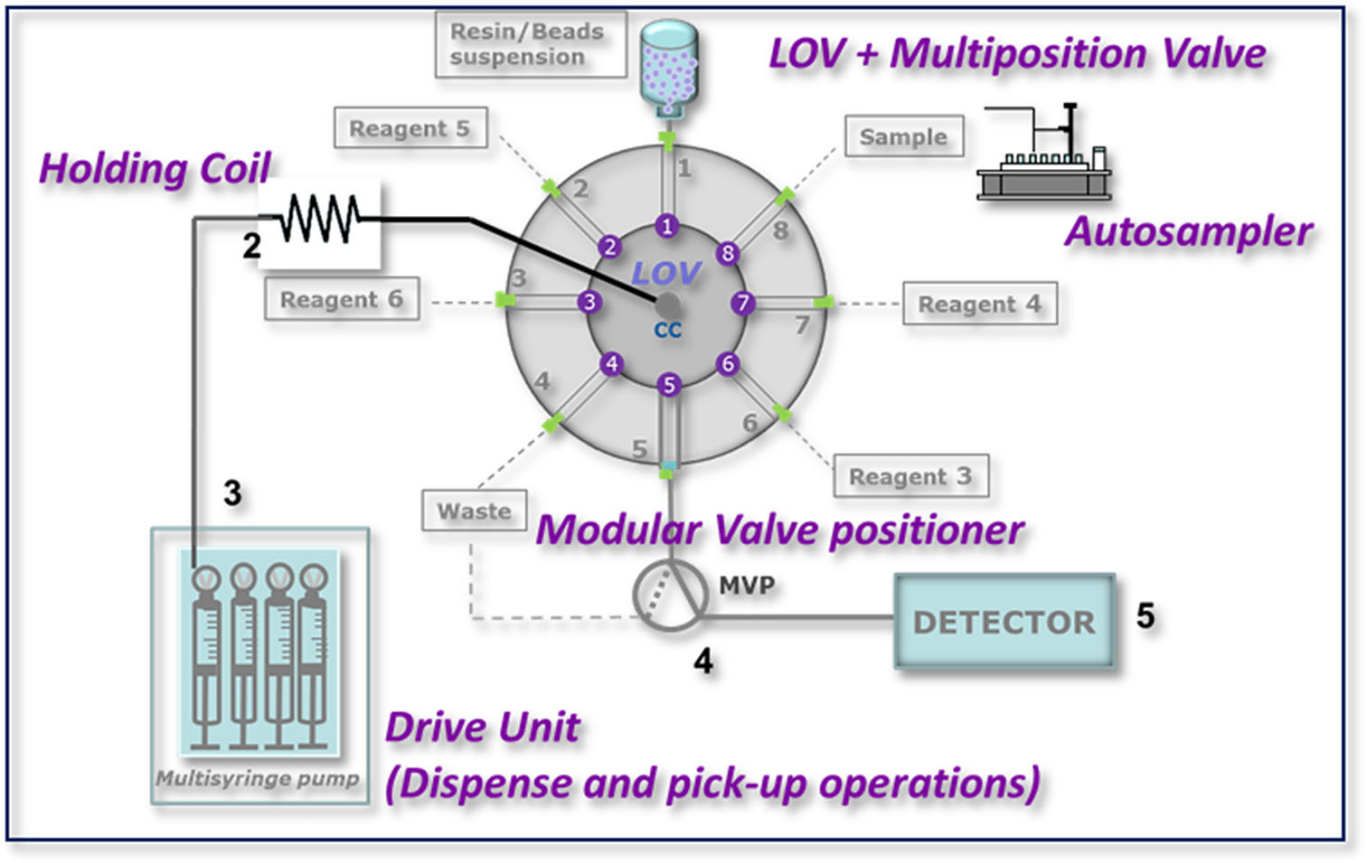
Peripheral port configuration

The central port of the valve in the system is connected to a 10 mL glass syringe (Hamilton Company Inc. Nevada, USA) via a 10 mL holding coil. The syringe is mounted in the MicroLab 600 syringe pump (Hamilton Company Inc. Nevada, USA). The syringe head has a three-way valve enabling multicommutation schemes (in: reservoir; out: system). The extraction resin (Sr-Resin, particle size 100–150 µm, 36 mg) is located in channel 1 of the LOV. A glass fiber filter holds the resin within the LOV channel allowing the solution flow. An autosampler (ESI, Omaha, Nebraska, USA) is connected to port 8 to allow quick processing of real samples. The peripheral port configuration was: port 7, eluent; port 1, resin suspension; port 2, washing solution; port 4, waste (Fig. 1). Port 5 is connected to a commutation valve (Hamilton Company Inc. Nevada, USA) which drives the flow coming from the LOV system in the desired manner (on: detector; off: waste; flush: resin exchange).

ICP-MS measurements were carried out in a double focusing sector field ICP-MS (Element 2, Thermo Finnigan MAT GmbH, Bremen, Germany). Element 2 is equipped with a self-aspiring micro-concentric nebulizer, a PC3 compact Peltier Cooled inlet system which incorporates the ESI cyclonic spray chamber, a Fassel torch and a 27 MHz generator. Isotopic measurements were performed using only electric scanning (E-scanning), at low (M/ΔM = 300) resolution settings. Instrumental settings and optimised measurement parameters for Sr isotopes are given in Table 1. The typical sensitivity at low resolution is 1.2–1.5 × 10^6^ cps per µg L^−1^ of strontium.

**Table 1:** HR-ICP-MS instrument settings and scanning conditions

Sample introduction system and instrumental operating conditions	
Nebuliser	0.1 mL min^−1^, self-aspiration mode
Spray chamber	PC^3^ peltier cooler
Sampling cone	Nickel
Skimmer cone	Nickel
Rf Power	1250
Plasma gas flow rate (L min^−1^)	15.5
Auxiliary gas flow rate (L min^−1^)	0.8
Nebuliser gas flow rate (L min^−1^)	1.0–1.2
Measurement conditions	
Resolution (10% valley definition)	Low, M/ΔM = 300
Acquisition mode	E-Scan
Magnet settling time (s)	0.300/0.0200
Magnet mass	
Mass range (amu)	
^86^Sr	85.766–86.052
^87^Sr	86.764–87.053
^90^Sr	89.757–90.057
Search window (%)	100
Integration window (%)	80
Sample time (s)	0.01
Sample per peak	20
Segment duration	0.2
Detection mode	EScan
Run & passes	160 × 1
Dead time correction (ns)	12

Online radioactivity detection was achieved using a β-RAM 5 flow detector (LabLogic, England), which model possesses an internal cocktail pump and can be fitted with flow cells of various types and sizes. All experiments were made using a 500 μL coiled Teflon flow cell placed in a fixed geometry between two photomultiplier tubes. Samples coming from the LOV are directed to the detector and mixed with the LSC Cocktail. The cocktail flow rate was set to 2 mL min^−1^. The detector counting parameters were controlled using Laura 4.2.8 (LabLogic Systems Ltd, Sheffield, UK) run on a desktop PC, connected to the detector via USB. Selection and triggering of the detector was performed via the developed software and an Arduino^®^ microprocessor [20].

For the preparation of all solutions, high-purity water (18.2 MΩ cm) from a Miliq-Element system designed for ultratrace analysis (Millipore, Milford, MA, USA) was used. Nitric acid, suprapur grade from Merck (Darmstadt, Germany), was purified using a quartz sub-boiling distillation unit. Both the water purification system and the sub-boiling distillation unit were operated in a clean room. Natural element standards were obtained from CPI international (Amsterdam, The Netherlands) as 1000 µg mL^−1^ stock standard solutions. A ^86^Sr standard solution of 10 mg L^−1^ (ESI, Omaha, Nebraska, USA), was used. A carrier-free radiostrontium standard solution containing 2000 Bq ml^−1^ was purchased from Eckert & Ziegler. Stock and working standards were prepared with Milli-Q deionized water.

## Programming-LabVIEW^®^ software interface

**Fig. 2:**
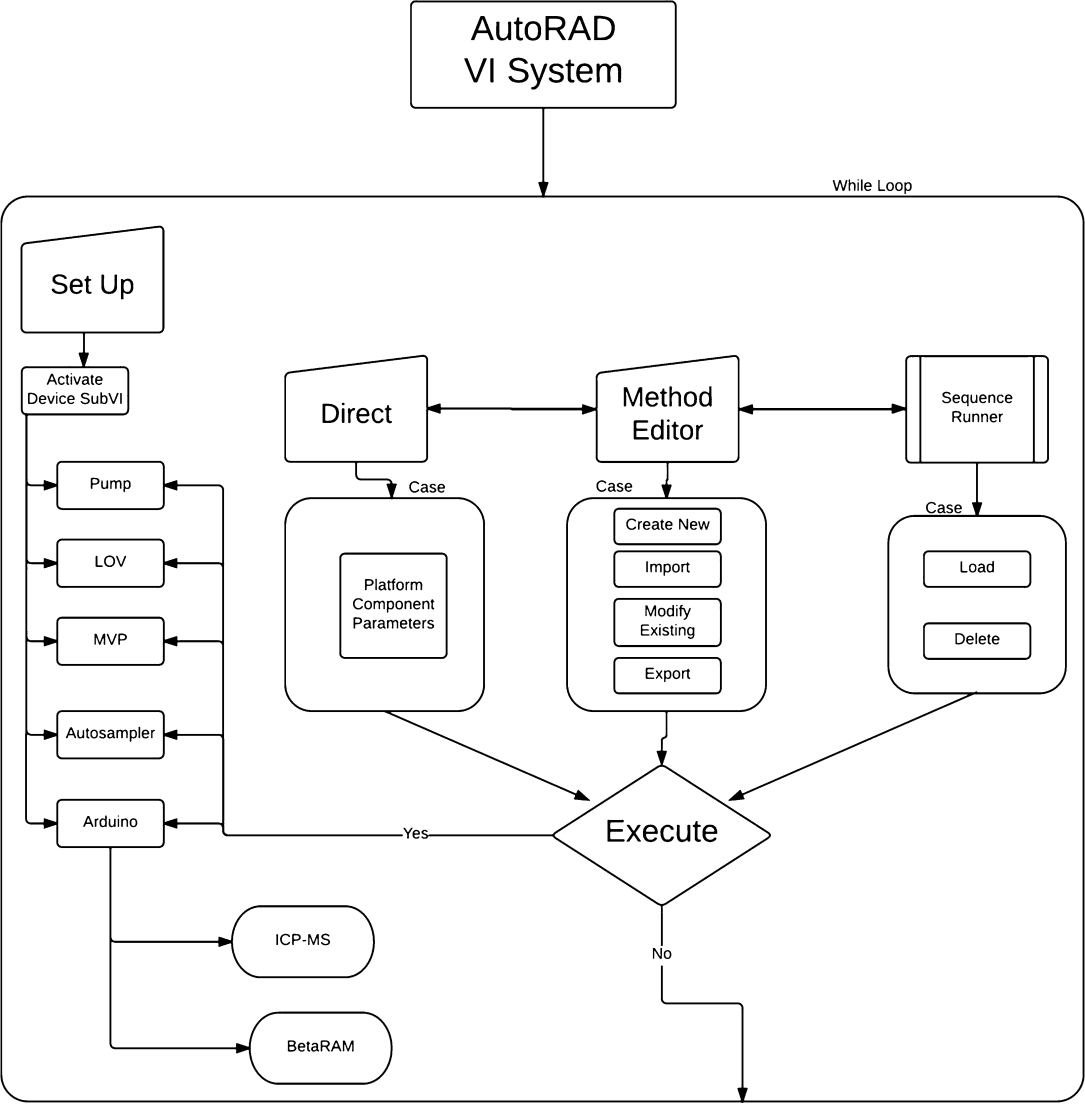
Schematic flow chart of the AUTORAD VI system

Since the program has to control the different hardware devices maintaining a user-friendly interface, all the actions required from the user are selected from drop-down menus to avoid typing errors. Moreover all subVIs and devices must be coordinated to perform all the analytical steps. This section shows the programming description of the main blocks, Fig indicates the operations performed by each subVI. The schematic flow chart of the AUTORAD VI system is represented in Fig. 2.

### Operational software

The software has been written with the SubVI function “Tab Control”, which allows the user to access different features without exiting the program. Tab structures execute actions depending on the user input. The welcome tab serves as a crossroads and allows the initialization of the different devices. Ready devices are shown with a green light. In the set-up tab, the selection of the devices to be used for the desired procedure can be performed.

**Fig. 3:**
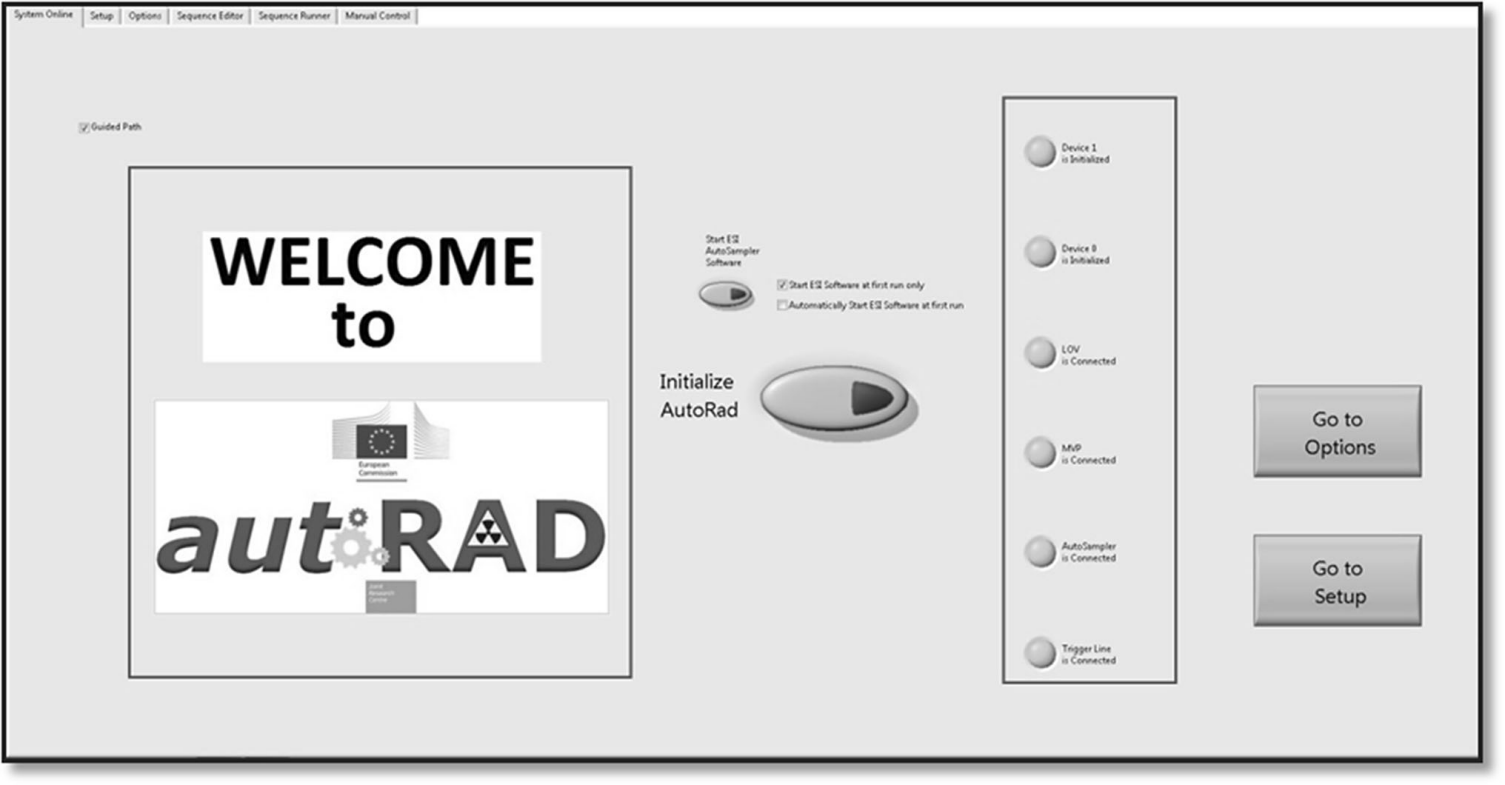
AUTORAD Main window graphical user interface, welcome menu

As soon as the software is running, the Hamilton^®^ Connect_0.vi (Hamilton Company Inc. Nevada, USA) sets up an IP connection between the computer and the syringe pumps, which are wired together over a Daisy Chain. To communicate with the devices (LOV, the Auto-sampler, the commutation valve as well as the in-house build Trigger Line), a VISA ConFig. Serial Port Node initialises a serial connection. Interfacing with the devices occurs via RS232 or USB, or conFig.d with company specific values. The Master loop then waits for further user action. Figure 3 show the software welcome tab.

### Programming for daily operation

Depending on the specific requirements, the user is able to select three different tabs “Direct”, “Method Editor” or “Sequence Runner” corresponding to software options. In the Editor tab, the user can create, modify or run single sequences whereas the Sequence Runner will allow programming of multiple sequences. Clearly, direct control allows the operator to perform single steps. The commands are chosen from a dropdown menu, making the software robust and user-friendly.

**Fig. 4:**
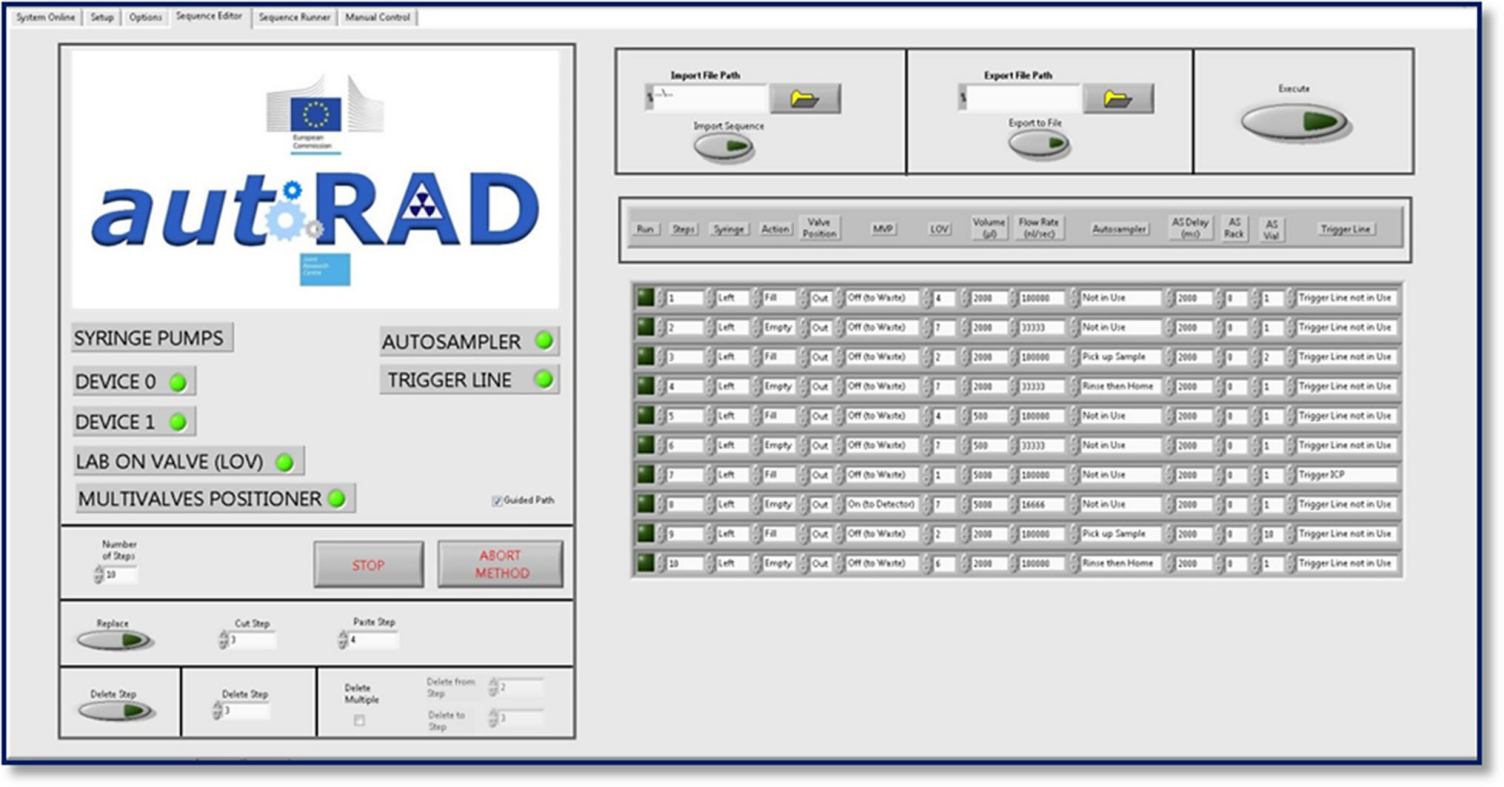
Graphical user interface (GUI), method editor tab

**Fig. 5:**
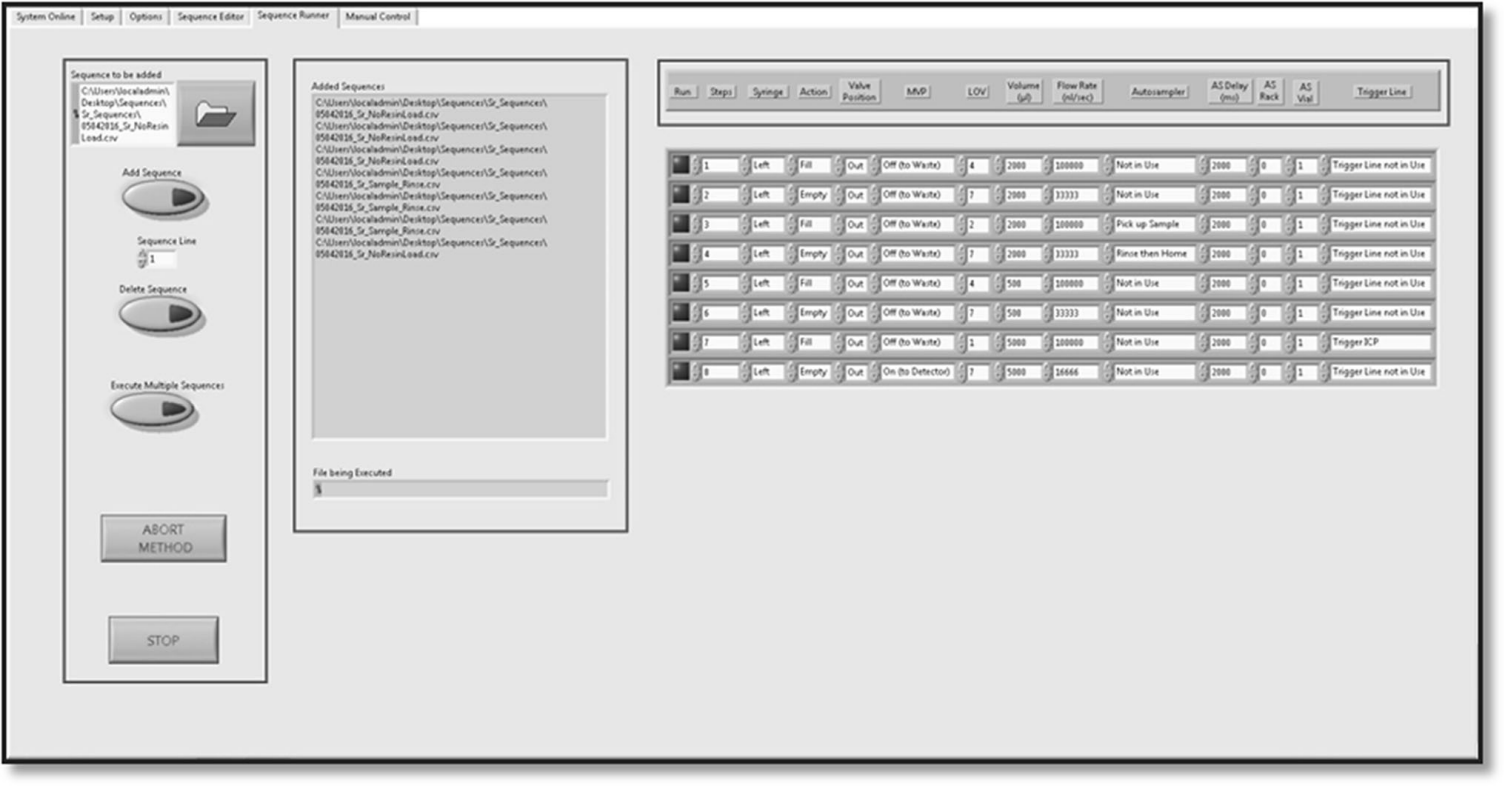
Graphical user interface, sequence runner tab

Figure 4 shows the operation panel in the “Method Editor” tab. The left-hand part on the screen displays information on the connected devices and the editing possibilities, such as Delete and Replace. On the right, administration buttons are integrated. The task of “Method Editor” is to create, modify, import or export sequences as well as running a work sequence. The data contained in the cluster can be easily accessed and ordered with functions such as Unbundle by Name. Modification of the data or execution through the devices can so be achieved. Figure 5 shows the operation panel in “Sequence Runner” allowing multiple sequences.

LabVIEW offers different possibilities to arrange and duplicate data. When simplicity is requested, the array function is a good choice to store and reproduce data. Arrays clone the input Object.

**Fig. 6:**
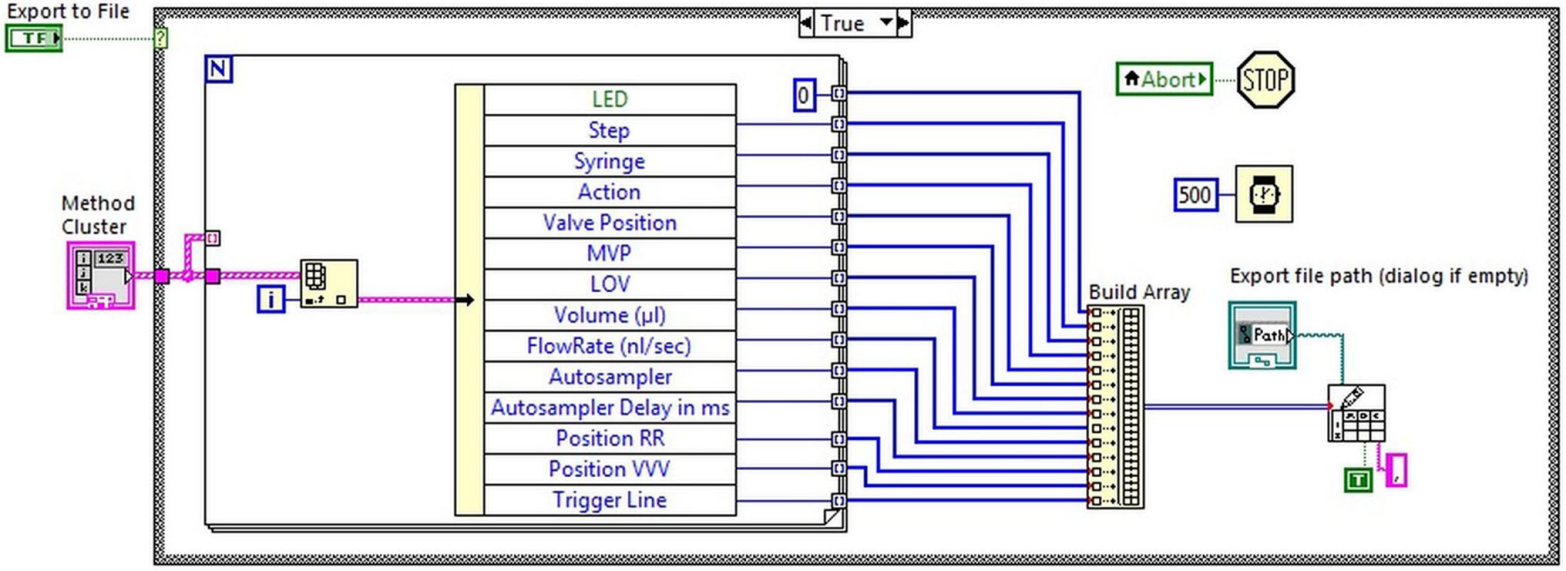
Application of a cluster

While Arrays are only able to store one Data Type, Clusters can contain multiple Data Types (e.g. Boolean, String, Integer) or even whole Arrays. Thus, they are more powerful, but as easy, as Arrays. The clear advantage of using Clusters is that being single objects, they are easy and safe to operate, making them the ideal tool to shift big amounts of data through the complex graphical block diagram. Combined with Enums (=enumerated values), standing for an operation governed by the devices, a user-friendly Menu is created. The user defines the number of steps in the GUI, and then the conFig.s for the devices operation. The Cluster is situated immediately under the buttons. Figure 6 shows an application of a cluster.

Reordering a step is achieved with the “Unbundle Cluster” function and “Insert Into Array” function, applied to the respecting steps.

The Export Button triggers the cluster to be input into a.csv file, which is then easily storable and accessible with various programs. The steps are ordered by category with Index Array Node, exported with Build Array Node into various Arrays and Export to Spreadsheet Node.

**Fig. 7:**
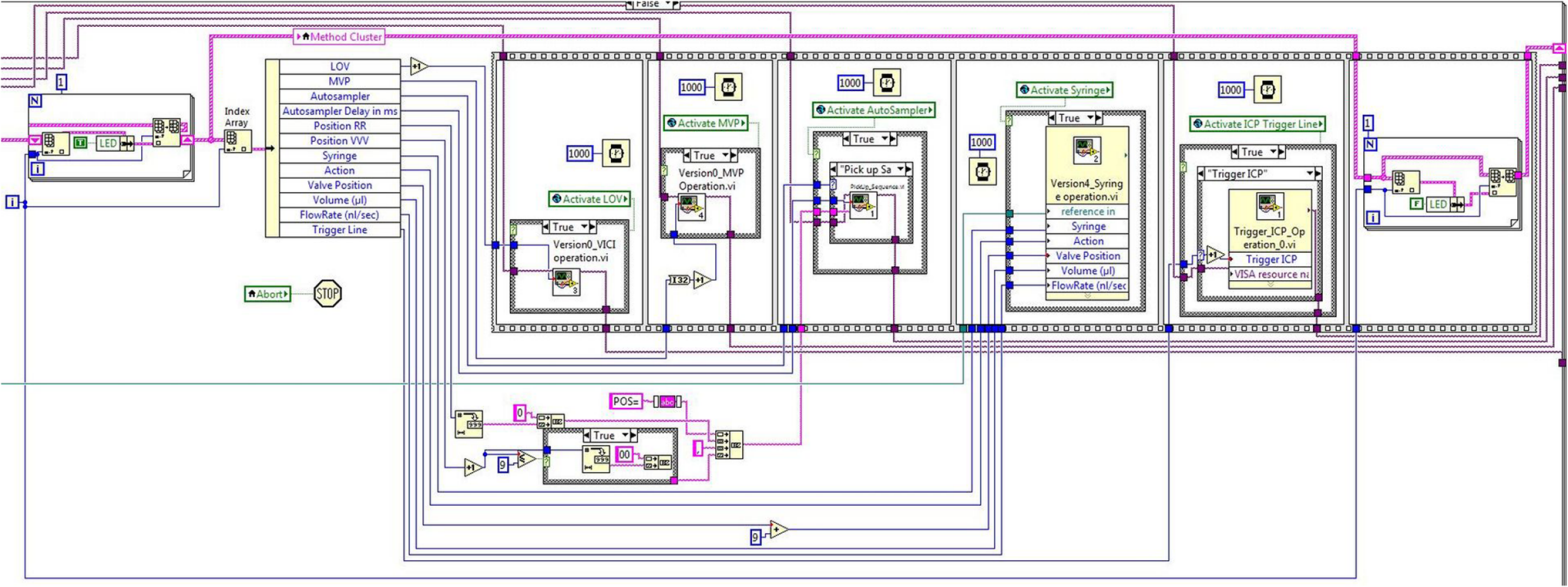
Block diagram of the developed software

When “Execute” is pressed, the unbundled data is input to a sequential frame, containing the subVI’s representing the devices, as schematically represented in the block diagram in Fig. 7.

### User friendliness

**Fig. 8:**
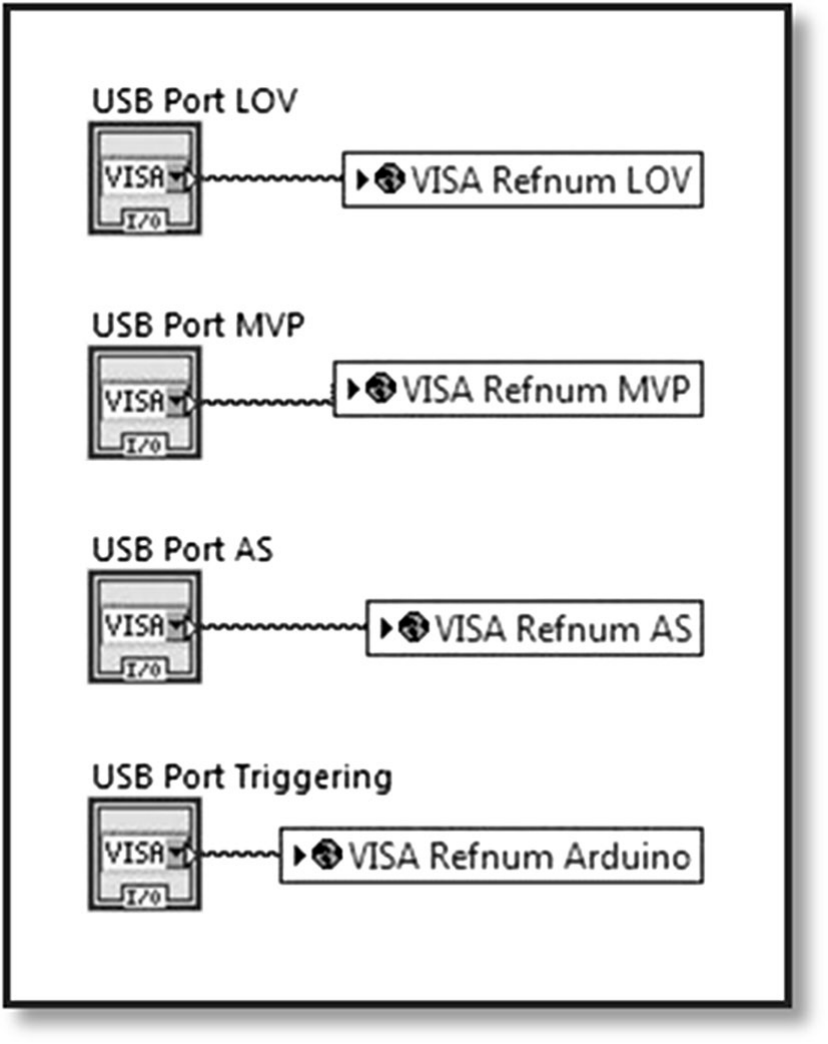
AUTORAD adaptable ports

The property nodes “Tip Strip” of the subjects are manipulated to gain an individually adaptable description. The options tab allows the user to configure the “Tip Strips” of every button and surface. To prevent offsetting, the ports read by AUTORAD are adaptable for every interfaced device (Fig. 8).

**Fig. 9:**
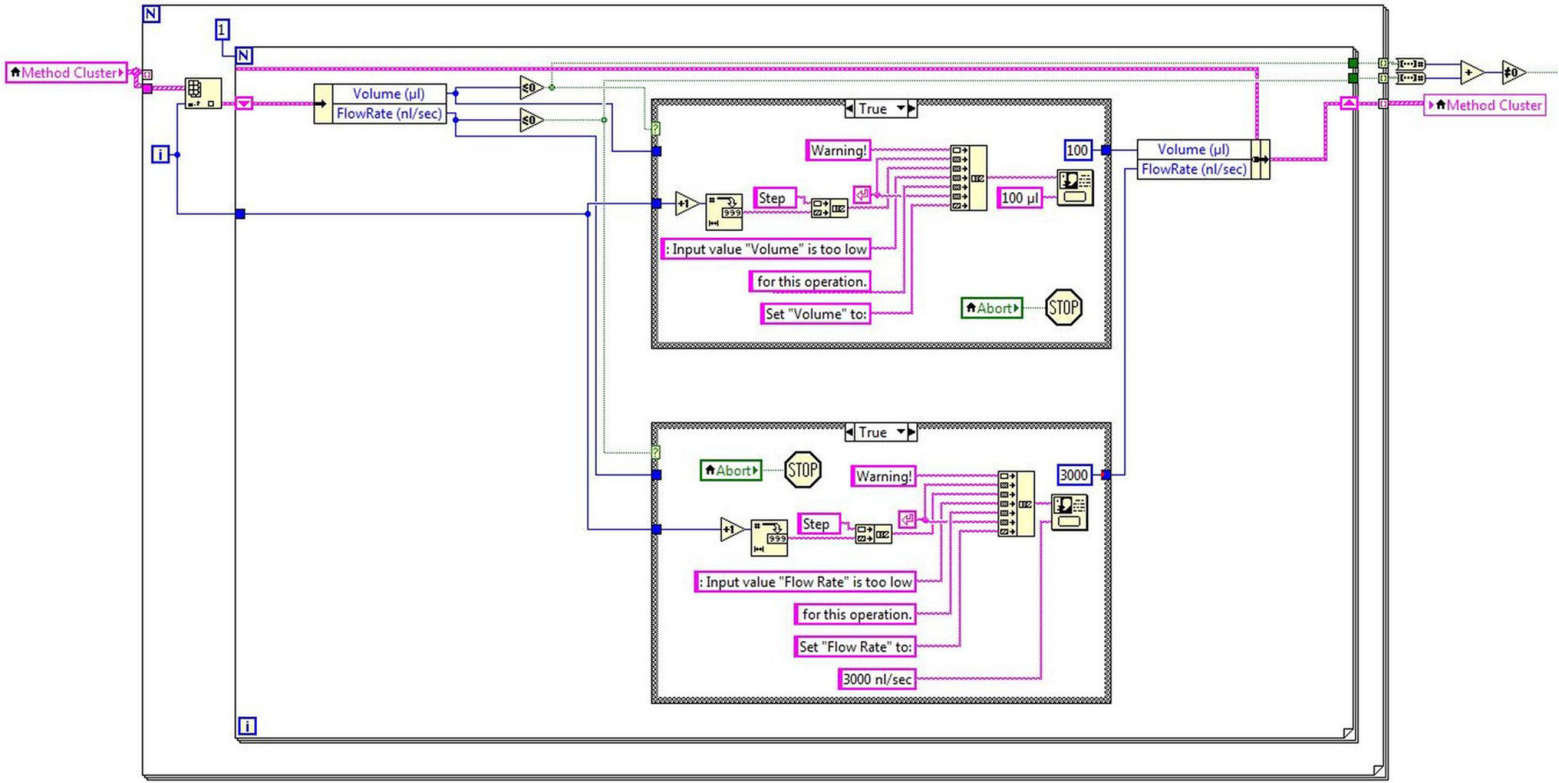
Monitoring algorithm

In order to prevent common errors, an algorithm governing the flow rate and syringe volume working ranges adapting to different models and suppliers, has been implemented. If needed, it asks the user to replace the critical value with an acceptable one. Programmatically this is achieved with the “Unbundling by Name” function and replacing the value (Fig. 9).

When starting the software, a specific sequence has to be followed to ensure a safe initialization of all connected devices. To respect this, the “Guided Path” algorithm hides the tabs buttons, suggesting a clear way to the desired end procedure.

## Results and discussion

The software described here has been tested and proven for the determination of strontium in standard samples at ultra-trace levels due to the importance of radiostrontium in nuclear waste management, decommissioning, toxicology and environmental monitoring issues. ^90^Sr and its daughter product ^90^Y are both pure β emitters, so determination of ^90^Sr by β counting requires separation from matrix constituents and interfering radionuclides prior to analysis. High specific activity and isobaric interferences represent a challenge in the spectrometric determination of ^90^Sr. The analysis of ^90^Sr is highly important, but due to the time consuming chemical separation, assumptions for the ^90^Sr/^137^Cs activity correlation are often taken for its estimation, rather than direct measurement [21].

Measurements on ^86^Sr were performed using the ICP-MS as detector, whereas the ^90^Sr determination was carried out with the β-RAM 5 flow detector. The Sr concentrations were 47.9 and 9.85 pg g^−1^, respectively. The complete operational sequence for strontium isolation, pre-concentration and on-line detection is listed in Table 2, and summarized as follows:

**Table 2:** Automated procedure for Sr separation, pre-concentration and detection

	Flow Rate (ml min^−1^)	LOV position	MVP
Resin loading			
(a) Loading beads into HC	6	1	Off
(b) Filling the column	1	5	Off
Conditioning of Sr-resin			
(a) Loading 2 mL of 4 mol L^−1^ HNO3 into HC	6	3	Off
(b) Rinsing 2 mL on the column	1	5	Off
Sample loading			
(a) Loading 1 mL sample into HC	6	8	Off
(b) Rinsing 1 mL on the column	1	5	Off
Elimination of interferences			
(a) Loading 0.5 mL of 4 mol L^−1^	6	3	Off
(b) Rinsing 0.5 mL on the column	1	5	Off
Elution of strontium			
(a) Loading 5 mL of MilliQ	6	7	Off
(b) Rinsing 5 mL on the column	1	5	On
Change of sample			
(a) Loading 1 mL of new sample into HC	6	8	Off
(b) Discarding 2 mL to the waste	6	4	Off
Resin replacing			
(a) Loading old resin into HC	6	5	Off
(b) Discarding old resin	6	4	Off
(c) Loading new resin into HC	6	1	Off
(d) Filling the column	1	5	Off

1. Loading of the resin: the column is automatically loaded with resin. First, the resin is loaded into the HC from the resin reservoir (port 1) which contains a saturated solution of the resin and dispensed at port 5 with V-off (to waste) to fill the column.
2. Conditioning of the Sr-Resin: the CC is connected to port 3 to aspirate 2 mL of 4 mol L^−1^ HNO_3_ into the HC. Then it moves to port 5 and the HNO_3_ is propelled toward the column at a flow rate of 6 mL min^−1^. V is deactivated (V-off, to waste).
3. Sample loading: once the column is ready, 1 mL of standard or sample (port 8) is dispensed toward the column (port 5) at a flow rate of 1 mL min^−1^.
4. Elimination of interferences: the CC is connected to port 3 to aspirate 0.5 mL of 4 mol L^−1^ HNO_3_ into the HC. Then it moves to port 5 and the HNO_3_ is propelled toward the column at a flow rate of 1 mL min^−1^.
5. Elution of Sr: at this point all the strontium retained on the column is eluted, with 5 mL of MilliQ water (port 7) loaded into the HC. V is activated (V-on) to propel the eluent through the column (port 5) at a flow rate of 1 mL min^−1^ to the detection system.
6. Change of sample: in order to avoid memory effects, 1 mL of the new sample is aspirated (port 8), and 2 mL are discarded toward waste (port 4).
7. Change of the resin: applicable, when required, depending on the sample matrix. The column is regenerated by replacing the resin automatically. First, the old resin is loaded into the HC and sent to waste (port 4), then new resin is loaded into the HC from the resin reservoir (port 1) which contains a saturated solution of the resin and dispensed at port 5 with V-off (to waste) to fill the column.

**Fig. 10:**
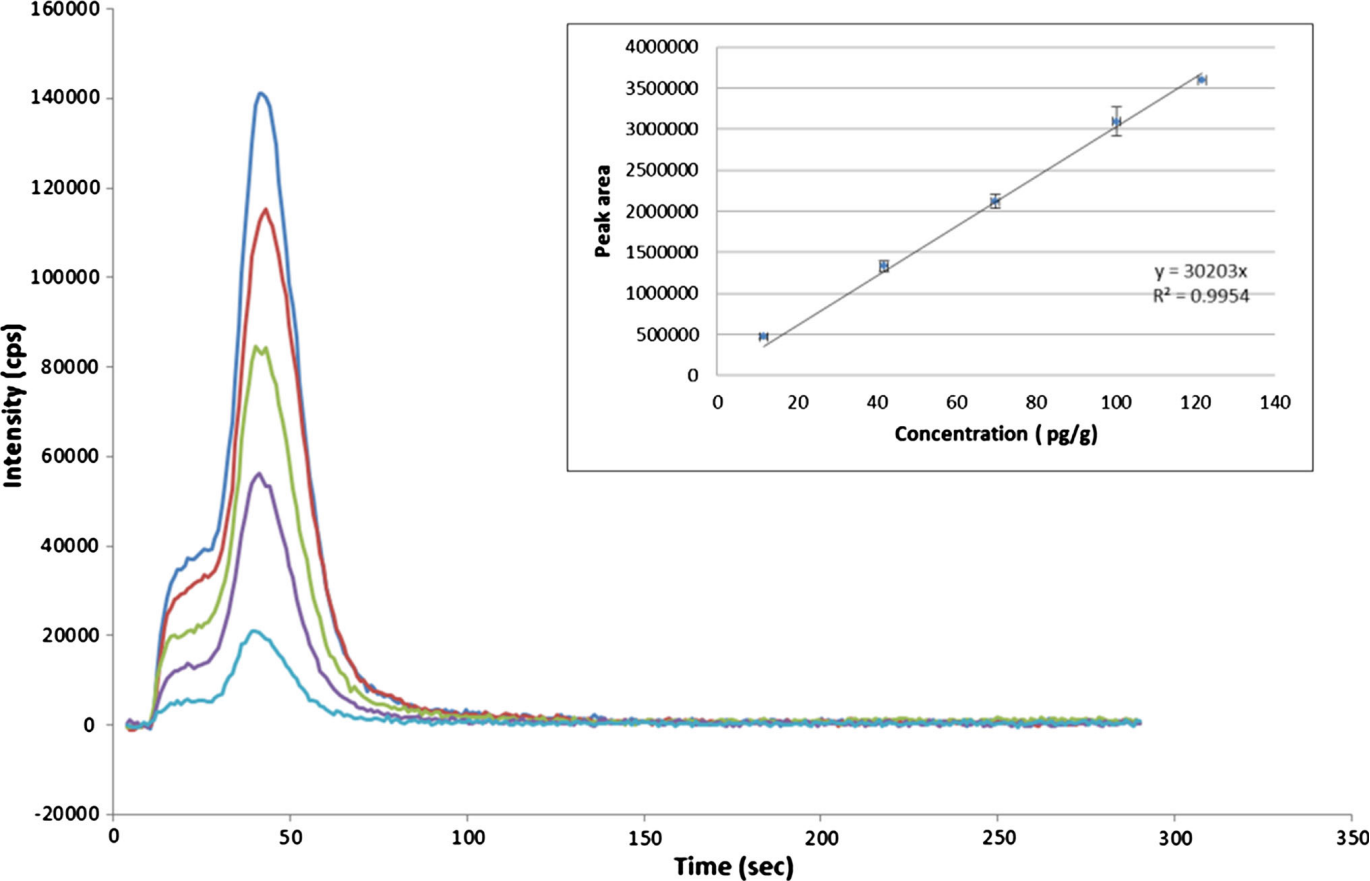
Sr elution peaks using ICP-MS as detector

The analytical performance of the AUTORAD platform was evaluated by considering the linearity, linear range, limit of detection (LOD) and repeatability. Figure 10 shows the corresponding ^86^Sr elution profiles using ICP-MS as well as the relationships between the peak area and ^86^Sr concentration. The linear regression yields a fit for purpose line which does not introduce extra uncertainty component. The LOD which is 2 pg g^−1^, was calculated by means of repeated measurements of the blank and according to Currie [22]. The repeatability of the method, based on the relative standard deviation of the peak area calculated on the basis of three runs is always less than 4% in this concentration interval (10–120 pg g^−1^). The elution profiles are in agreement with those reported for radiostrontium using low pressure devices [13, 23] and have been blank subtracted. The shoulder witnessed in the elution profiles is most likely an artefact due to a less than optimal packing of the column, simply due to the automated AUTORAD configuration. Nevertheless, it has no impact on the analytical performance parameters.

**Fig. 11:**
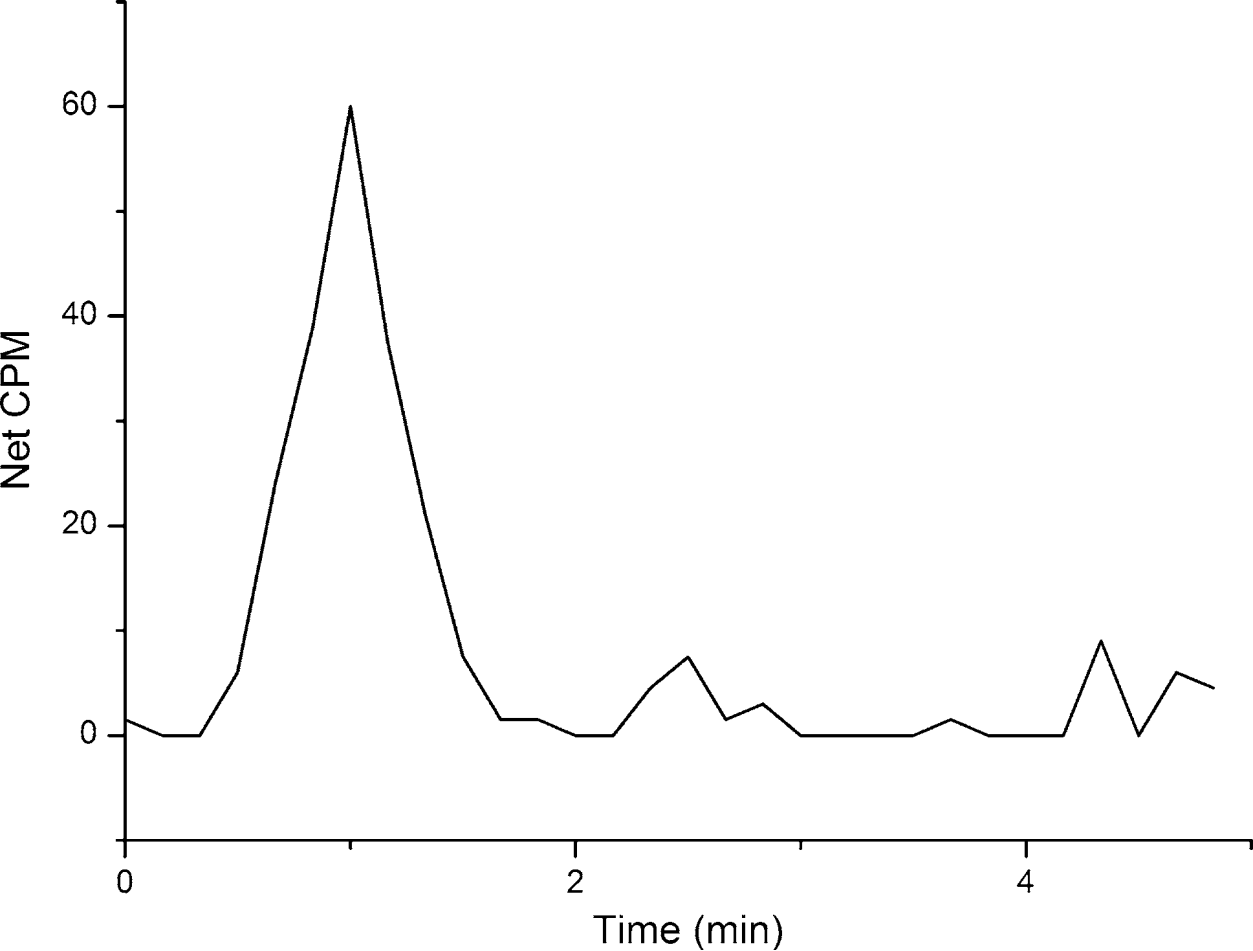
36 Bq ^90^Sr (7 pg g^−1^) elution peak using β-RAM 5 as detector. Residence time in the coil was 10 s. The LOD within this configuration has been determined to be 2 Bq (0.4 pg g^−1^) according to Currie [22]

Figure 11 shows the ^90^Sr profile using the the BetaRAM 5 radio flow detector demonstrating the feasibility of AUTORAD software for the separation and simultaneous detection of ^90^Sr in aqueous samples. The optimal counting window for ^90^Sr with the β-RAM flow scintillation analyzer, and the determination of several counting parameters have still to be optimized. The stopped-flow technique, is being investigated as it could improve the method sensitivity by extending, indefinitely, the residence time of the largest part of the sample zone within the flow cell, allowing a statistically meaningful number of counts to accumulate before the sample is permitted to exit the detector.

## Conclusions

A fully automated AUTORAD platform operated by homemade LabVIEW^®^-based software has been developed and implemented. Its applicability to real samples was tested with Sr standard samples. The platform is versatile and is able to operate with different detectors. The simple and logical structure of the software makes it robust, user-friendly and suitable for on-site measurements.

The AUTORAD platform is a multi-purpose tool for performing safe, faster, less laboratory intensive measurements and is expected to be used as a powerful and convenient tool for the chemical separation and measurement of radioisotopes. Thus, monitoring operations at-site or in situ measurements will be more effective than traditional sampling and manual laboratory analyses.

Nuclear decommissioning, nuclear site remediation and monitoring R&D needs to include measurement technology improvements to optimize and reduce intrusive sampling applications as they are costly, time consuming and can lead to unnecessary worker radiation exposure.
